# Systematic profiling reveals hepatic immune and metabolic dysregulation in DNASE1L3-deficient mice

**DOI:** 10.1016/j.isci.2025.114198

**Published:** 2025-11-22

**Authors:** Liangchen Lei, Jiaxiu Yu, Bo Zhang, Pengpeng Liu, Zhuo Meng, Youai Song, Jianwei Lan, Binjie Li, Quanyan Liu

**Affiliations:** 1 Department of General Surgery, Tianjin Medical University General Hospital, Tianjin 30052, P.R. China

**Keywords:** cell biology, immunology, molecular biology, omics

## Abstract

Deoxyribonuclease 1-like 3 (DNASE1L3) is a secreted endonuclease essential for degrading extracellular DNA and maintaining immune tolerance, but its role in hepatic immune-metabolic regulation remains unclear. Using multi-omics analyses combined with immunophenotyping, we demonstrate that *Dnase1l3*-deficient (knockout, KO) mice exhibit disrupted myeloid differentiation, Kupffer cell M1 polarization (M1), and progressive hepatic steatosis. Integrated transcriptomic, proteomic, and metabolomic profiling revealed activation of damage-associated molecular pattern (DAMP) sensing, endoplasmic reticulum stress, redox imbalance, ferroptosis susceptibility, and lipid metabolic reprogramming. Whole-genome resequencing further identified chromosome 4 mutation hotspots linked to iron metabolism, oxidative stress, and inflammation. These findings establish DNASE1L3 as a key regulator of hepatic immune-metabolic homeostasis and suggest that its deficiency drives a pathological cascade involving pattern recognition receptor activation, endoplasmic reticulum stress, and ferroptosis, ultimately leading to non-alcoholic fatty liver disease (NAFLD). This study provides a mechanistic framework for nucleic acid-driven immunometabolic dysregulation in chronic liver disease.

## Highlights

• DNASE1L3 loss impairs myeloid differentiation and drives spontaneous hepatic steatosis
• Transcriptomics reveal DAMP signaling activation and inflammatory reprogramming in liver
• DNASE1L3 deficiency triggers Kupffer cell ER stress and immunometabolic remodeling
• Multi-omics profiling links redox imbalance and ferroptosis to hepatic metabolic dysfunction

## Graphical abstract

**Figure:**
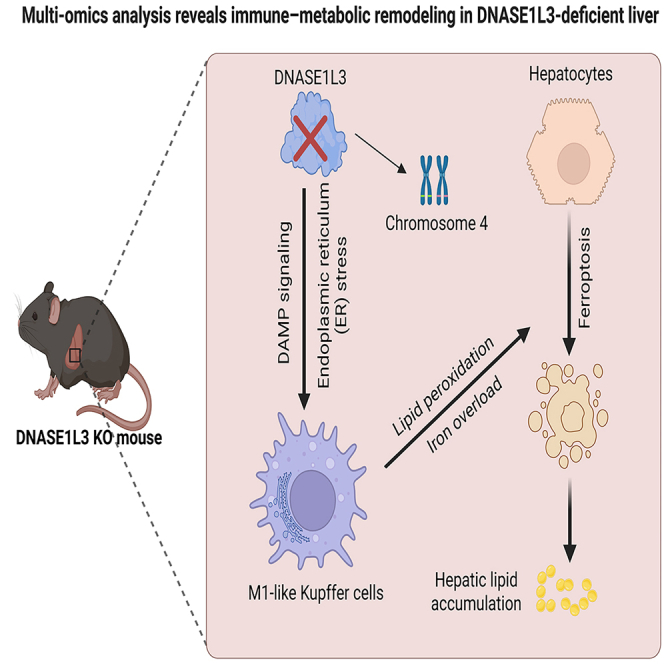

## Introduction

DNASE1L3 is a secreted endonuclease predominantly expressed by monocyte-macrophage lineage cells, with particularly high levels observed in the liver.^1^^,^^2^ Recent work has further identified DNASE1L3 expression in hepatic sinusoidal endothelial cells, indicating a broader cellular distribution within the liver.^3^ This enzyme plays a pivotal role in maintaining immune tolerance by degrading extracellular cell-free DNA (cfDNA) released during apoptosis or necrosis.^4^ Loss-of-function mutations in DNASE1L3 may result in pathological cfDNA accumulation, which has been associated with sterile inflammation and immune dysregulation characteristic of systemic autoimmune disorders such as systemic lupus erythematosus (SLE).^5^^,^^6^^,^^7^ Furthermore, DNASE1L3 regulates plasmacytoid dendritic cell expansion and B cell activation, underscoring its broader role in immune homeostasis.^8^^,^^9^ Emerging evidence suggests that DNASE1L3 deficiency exacerbates autoantibody production and dysregulated immune responses, reinforcing its critical function in immune balance.

Beyond systemic immunity, DNASE1L3 may have organ-specific roles, particularly in the liver—a central hub for metabolic and immune regulation.^10^ Recent studies highlight its potential in mitigating sterile inflammation, such as through NOD-, LRR-, and pyrin domain-containing protein 3 (NLRP3) inflammasome suppression and interleukin-1 beta (IL-1β) reduction via cfDNA degradation.^11^ These mechanisms are highly relevant to chronic liver diseases, including NAFLD, a global health challenge characterized by lipid metabolism disorders, inflammation, and immune-metabolic dysregulation.^12^^,^^13^ NAFLD progression involves impaired Kupffer cell (KC) activity, the liver’s resident macrophages, which are critical in maintaining immune-metabolic homeostasis.^14^ KCs undergo metabolic reprogramming and polarization shifts that exacerbate oxidative stress and inflammation, driving NAFLD pathogenesis.

DNASE1L3’s hepatic relevance extends to cancer biology, where its downregulation correlates with poor prognosis, increased angiogenesis, and immune suppression in hepatocellular carcinoma (HCC).^15^ Restoring DNASE1L3 expression may inhibit tumor progression by stabilizing the hepatic microenvironment, though the mechanistic interplay between DNASE1L3, KCs, and metabolic dysfunction remains poorly understood.^16^^,^^17^

To address these gaps, we generated a global DNASE1L3 knockout (KO) mouse model and combined immune phenotyping with multi-omics approaches—including transcriptomics, proteomics, and metabolomics—to systematically evaluate the biological effects of DNASE1L3 deficiency in the liver.^18^^,^^19^ This strategy aligns with cutting-edge multi-omics frameworks advocated for NAFLD assessment, which combine machine learning and molecular profiling to uncover disease mechanisms. Our study focuses on cfDNA signaling, KC polarization, hepatic oxidative stress, and metabolic reprogramming, offering a comprehensive analysis of DNASE1L3’s impact on immune-metabolic homeostasis. By elucidating these multilayered molecular alterations, we aim to provide mechanistic insights into NAFLD progression and identify therapeutic targets for this increasingly prevalent disease.

## Results

### DNASE1L3 deficiency impairs myeloid progenitor differentiation and induces spontaneous hepatic steatosis

**Figure 1:**
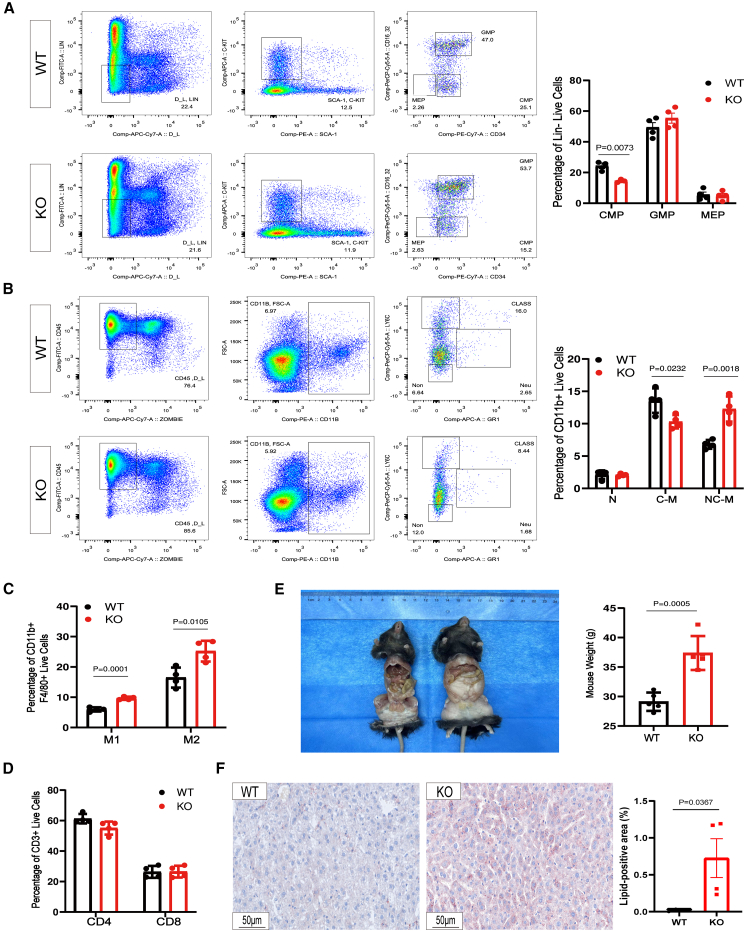
DNASE1L3 deficiency impairs myeloid progenitor development and induces spontaneous fatty liver phenotype in aged mice (A) Flow cytometric analysis of bone marrow cells from 8-week-old WT and KO mice. After exclusion of debris and doublets, live Lin-cells were gated, and myeloid progenitor subsets were identified within the c-Kit+Sca-1- population based on CD34 and CD16/32 expression: CMP (CD34^+^CD16/32ˆlow), GMP (CD34^+^CD16/32ˆhigh), and MEP (CD34^−^CD16/32ˆlow). Right image shows quantification of each subset (*n* = 4 per group). (B) Splenic monocyte subsets were analyzed by flow cytometry. Classical monocytes (C-M): CD11b+GR1-Ly6Cˆhigh; non-classical monocytes (NC-M): CD11b+GR1-Ly6C-; intermediate monocytes (N): CD11b+GR1-Ly6Cˆint. Neutrophils were defined as CD11b+Ly6CˆintLy6G+ or GR1+ (*n* = 4 per group). (C) Quantification of M1 (CD86^+^) and M2 (CD206+) macrophages in splenic tissues, showing a significant increase in M1-like macrophages in KO mice (*n* = 4 per group). (D) Quantification of CD4^+^ and CD8^+^ T cells gated from splenic lymphocytes (*n* = 4 per group). (E) Representative gross morphology of 50-week-old WT and KO mice. KO mice exhibited apparent hepatomegaly and increased visceral fat accumulation (left). Quantification of body weight revealed a significant increase in KO mice compared with WT controls (right; *n* = 5; *p* = 0.0005). (F) Representative Oil Red O staining of liver sections from WT and KO mice showing increased hepatic lipid deposition in DNASE1L3-deficient mice (left). Quantification of lipid-positive area revealed a significant elevation in KO livers compared with WT controls (right; scale bars, 50 μm; *n* = 4 per group). Data are presented as mean ± SEM. Statistical comparisons were performed using unpaired two-tailed Student’s *t* test. Exact *p* values are indicated; *p* < 0.05 was considered statistically significant.

To investigate the role of DNASE1L3 in immune homeostasis, we performed flow cytometric analysis of bone marrow hematopoietic progenitor cells in 8-week-old KO and wild-type (WT) mice. The frequency of common myeloid progenitors (CMPs) was significantly reduced in KO mice (Figure 1A), consistent with the critical role of DNASE1L3 in early hematopoietic regulation. In contrast, granulocyte-monocyte progenitors (GMPs) and megakaryocyte-erythroid progenitors (MEPs) were unaltered (Figure 1A), indicating stage-specific disruption of myelopoiesis.

Further analysis of splenic monocyte subsets revealed a marked decrease in classical monocytes (CD11b^+^GR1^−^Ly6C^high^) and a significant increase in non-classical monocytes (CD11b^+^GR1^−^Ly6C^−^) in KO mice (Figure 1B), suggesting altered monocyte subset composition. Given that monocytes differentiate into tissue macrophages, we examined splenic macrophages and observed a significant increase in CD11b^+^F4/80^+^ macrophages in KO mice (Figures 1C and S1A). These findings are consistent with a skewed myeloid differentiation toward macrophages in the absence of DNASE1L3, suggesting a potential role for DNASE1L3 in maintaining myeloid lineage balance. Analysis of splenic CD4^+^ and CD8^+^ T cells showed no differences between genotypes (Figures 1D and S1B), highlighting the myeloid-specificity of DNASE1L3-dependent immune dysregulation.

Considering the potential for progressive phenotypes in DNASE1L3-associated immune dysregulation, we evaluated aged mice at 50 weeks.^6^ *Dnase1l3*-deficient mice exhibited significantly increased body weight and developed pronounced hepatomegaly accompanied by hepatic lipid accumulation (Figure 1E). Histological analysis revealed widespread macrovesicular steatosis in the livers of KO mice, whereas WT livers retained normal hepatic architecture (Figure 1F). These results suggest that DNASE1L3 deficiency may contribute to the development of spontaneous NAFLD, even in the absence of dietary or pharmacologic triggers.

Collectively, DNASE1L3 deletion is associated with impaired myeloid progenitor differentiation, peripheral monocyte imbalance, and macrophage expansion. These immune alterations may promote hepatic immune infiltration and chronic inflammation, potentially contributing to the development of a pro-NAFLD microenvironment. Our findings support a potential role for DNASE1L3 in bridging immune homeostasis and hepatic metabolic regulation.

### Transcriptomic profiling reveals DAMP signaling activation and proinflammatory gene signatures in DNASE1L3-deficient livers

**Figure 2:**
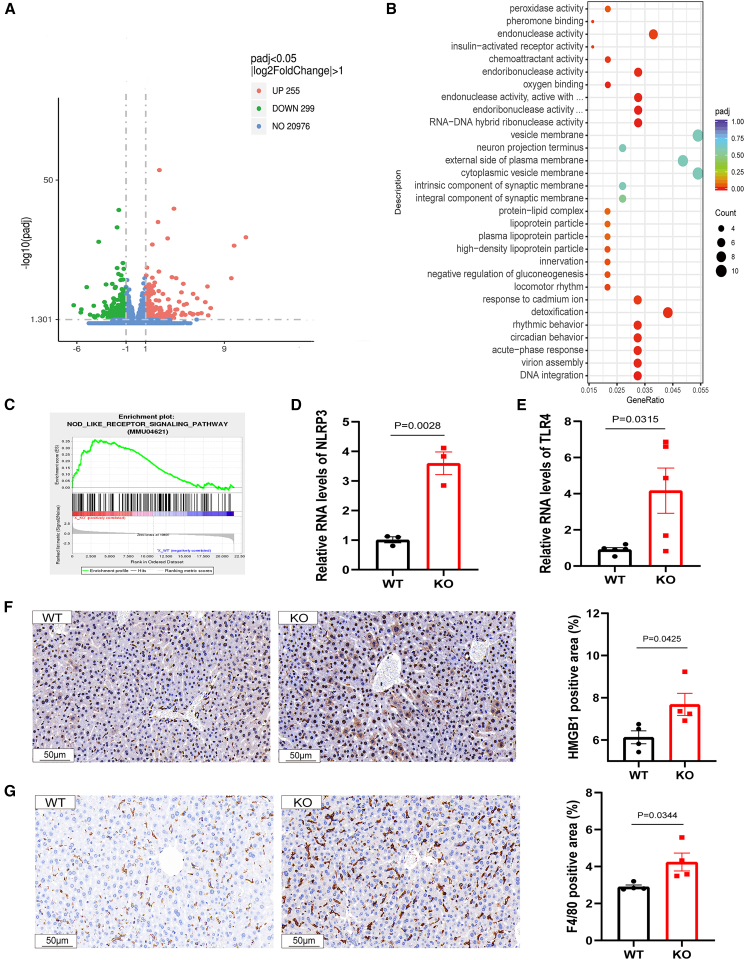
DNASE1L3 deficiency induces hepatic pro-inflammatory transcriptional reprogramming and inflammatory activation (A) Volcano plot of DEGs in liver tissues from WT and KO mice (*n* = 3 per group). A total of 255 genes were significantly upregulated (green) and 299 downregulated (red) in KO livers (adjusted *p* < 0.05, |log2 fold change| > 1). Non-significant genes are shown in blue. (B) GO enrichment analysis of the top 30 upregulated DEGs revealed significant enrichment in terms related to oxidoreductase activity, membrane protein complexes, acute-phase response, and endonuclease activity, suggesting activation of innate immune and stress-response pathways. (C) GSEA demonstrated significant enrichment of the NOD-like receptor signaling pathway in KO livers (NES = 1.59, nominal *p* = 0.003). (D and E) Quantitative RT-PCR analysis showing elevated hepatic mRNA levels of Nlrp3 (*p* = 0.0028) and Tlr4 (*p* = 0.0315) in KO mice compared with WT controls (*n* = 3–5 per group). (F) Representative IHC staining of HMGB1 in liver sections from WT and KO mice (left). KO livers showed stronger HMGB1 expression and cytoplasmic translocation. Quantification of HMGB1-positive area confirmed a significant increase in KO mice (right; *p* = 0.0425; scale bars, 50 μm; *n* = 4 per group). (G) Representative IHC staining of the macrophage marker F4/80 in liver sections (left). Quantification revealed significantly higher F4/80-positive area in KO livers than in WT controls (right; *p* = 0.0344; scale bars, 50 μm; *n* = 4 per group). Data are presented as mean ± SEM. *p* values were determined by unpaired two-tailed Student’s *t* test. Exact *p* values are indicated; *p* < 0.05 was considered statistically significant.

To elucidate the molecular mechanisms underlying DNASE1L3 deficiency-induced fatty liver development, we performed transcriptomic profiling of liver tissues from 8-week-old KO and WT mice, thereby minimizing confounding effects of aging (Figure S1C). A total of 554 differentially expressed genes (DEGs) were identified (255 upregulated and 299 downregulated) (Figure 2A), indicating early transcriptomic reprogramming.

Gene ontology (GO) enrichment analysis revealed that upregulated genes were associated with nucleic acid processing functions (e.g., endonuclease activity), vesicular and lipid-related compartments (e.g., vesicle membrane, high-density lipoprotein particle), and biological processes linked to inflammation and metabolic stress (e.g., acute-phase response, negative regulation of gluconeogenesis, and response to metal ions) (Figure 2B). These findings suggest that DNASE1L3 deficiency not only perturbs metabolic pathways but also primes the liver for inflammatory activation.

Consistent with this, gene set enrichment analysis (GSEA) demonstrated significant enrichment of innate immune pathways, particularly NOD-like receptor and Toll-like receptor signaling, indicating activation of pattern recognition receptors (PRRs) (Figures 2C, S1D, and S1E). A trend toward T cell receptor signaling enrichment was also observed (Figure S1F), suggesting potential crosstalk between innate and adaptive immunity.

To validate these transcriptomic findings, qPCR analysis demonstrated significant upregulation of key DAMP/PRR-related genes in KO livers, including *Nlrp3* (*p* = 0.0028), *Tlr4* (*p* = 0.0315), and *Zbp1* (*p* = 0.0076) (Figures 2D, 2E, and S1G).

At the protein level, immunohistochemistry (IHC) revealed increased expression and cytoplasmic translocation of high-mobility group box 1 (HMGB1), a prototypical DAMP, in KO livers compared with WT controls (Figure 2F).^20^ Quantitative IHC analyses further showed significantly elevated hepatic expression of proinflammatory cytokines and macrophage markers, including tumor necrosis factor (TNF) (*p* < 0.0001), interleukin-6 (IL-6) (*p* = 0.0105), monocyte chemoattractant protein-1 (MCP-1) (*p* = 0.0031), and F4/80 (*p* = 0.0344), in KO mice (Figures 2G and S1H–S1J).

Together, transcriptomic and immunohistochemical analyses revealed that DNASE1L3 deficiency was associated with the establishment of a proinflammatory hepatic program. This was reflected by enrichment of PRR-related signaling pathways, upregulation of inflammatory mediators, and evidence of metabolic stress at the transcriptomic level, as well as increased hepatic expression of HMGB1, TNF, IL-6, MCP-1, and F4/80 detected by IHC.

### DNASE1L3 deficiency induces proinflammatory polarization and immunometabolic reprogramming of KCs

Building on previous observations that KO mice display hepatic lipid metabolic disruption and heightened inflammatory activation, accompanied by enrichment of DAMP-sensing pathways and immune cell infiltration, we next examined the impact of DNASE1L3 deficiency on the immunophenotype and metabolic features of KCs—liver-resident macrophages that function as key innate immune sensors for fatty acids and microbial-derived DAMPs through a broad repertoire of PRRs.^21^^,^^22^

**Figure 3:**
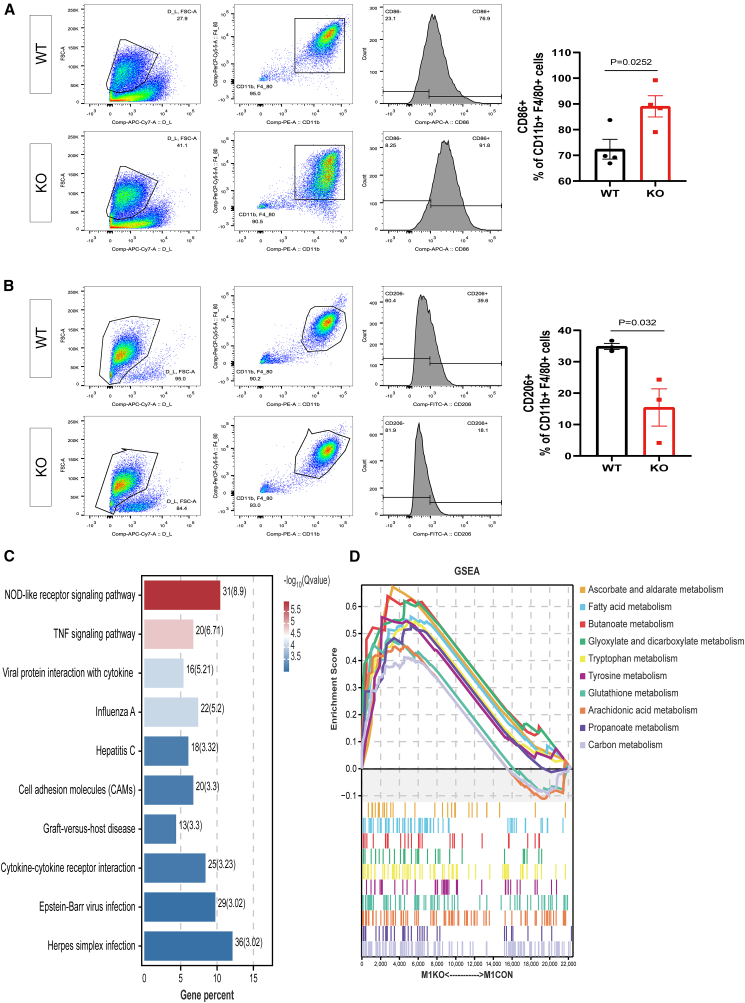
DNASE1L3 deficiency promotes M1 polarization of KCs and activates immune-related signaling pathways (A and B) Flow cytometric analysis of liver-resident KCs (CD11b+F4/80+) from WT and KO mice. (A) The proportion of M1-polarized KCs (CD86^+^) was significantly increased in the KO group (*p* = 0.0252; *n* = 4 per group). (B) In contrast, the proportion of M2-polarized KCs (CD206+) was significantly decreased in KO mice (*p* = 0.032). Bar graphs show mean ± SEM (*n* = 3 per group). Statistical comparisons were performed using unpaired two-tailed Student’s *t* test. (C) KEGG pathway enrichment analysis of upregulated genes in KO KCs showed significant enrichment in inflammatory and pathogen-response pathways, including the NOD-like receptor signaling pathway, TNF signaling, and cytokine-cytokine receptor interaction. Pathways are ranked by –log_10_ (*p* value), indicating the significance of enrichment (*n* = 3 per group). (D) GSEA revealed downregulation of key metabolic pathways in KO KCs, including fatty acid metabolism, glutathione metabolism, pyruvate metabolism, and tryptophan metabolism, indicating immunometabolic reprogramming associated with proinflammatory activation. For pathway analysis, a cutoff of *p* < 0.05 was used for significance. GSEA enrichment was assessed based on both nominal *p* values and FDR *q* values (see STAR Methods for details).

We examined DNASE1L3 expression across hepatic cell populations under NAFLD conditions and observed that its expression was significantly reduced in KCs (Figure S2A). Flow cytometric analysis further showed that lipopolysaccharide (LPS) stimulation significantly increased the proportion of M1-polarized KCs in *Dnase1l3*-deficient mice compared with WT controls (*p* = 0.0252), whereas interleukin-4 (IL-4)-induced polarization toward the alternatively activated M2 macrophage phenotype (M2) was reduced (*p* = 0.032) (Figures 3A and 3B).

To elucidate the underlying molecular mechanisms, we performed RNA sequencing of KCs exposed to M1-polarizing stimuli *in vitro*. Kyoto Encyclopedia of Genes and Genomes (KEGG) pathway enrichment analysis of DEGs revealed significant activation of multiple inflammatory signaling pathways in KO KCs. Notably, the NOD-like receptor signaling pathway was among the top-ranked pathways (gene count = 31, −log_10_(*q*) = 8.9), along with upregulation of TNF signaling, cytokine-cytokine receptor interaction, and chemokine signaling pathways, indicating a robust inflammatory activation state (Figure 3C). Co-enrichment of the cell adhesion molecules (CAMs) pathway and chemokine-associated modules further suggests that KO KCs may acquire enhanced immune recruitment capacity, thereby amplifying intrahepatic inflammation.

GSEA further revealed significant upregulation of lipid metabolism pathways—including fatty acid metabolism and arachidonic acid metabolism—in KO KCs (Figure 3D). Antioxidant defense pathways, particularly glutathione metabolism, were also positively enriched, reflecting intrinsic counter-regulatory responses to oxidative stress. The enrichment of carbon, glycine, and pyruvate metabolism pathways is consistent with coordinated reprogramming of immune activation and energy metabolism.

Together, these findings suggest that DNASE1L3 deficiency is associated with M1-like pro-inflammatory polarization of KCs and may contribute to the concurrent activation of inflammatory signaling and metabolic remodeling. This dual-state reprogramming supports a potential role for KCs as key hepatic effectors linking innate immune dysregulation to metabolic liver disease pathogenesis.

### DNASE1L3 deficiency induces endoplasmic reticulum stress in KCs and orchestrates inflammatory and metabolic dysregulation

Our previous findings revealed that DNASE1L3 deficiency promotes M1-like proinflammatory polarization of KCs, accompanied by widespread activation of immune and metabolic pathways. However, the upstream regulatory mechanisms driving this immunometabolic shift remain incompletely understood.

Multi-omics analyses suggested enhanced oxidative phosphorylation in KCs from *Dnase1l3*-deficient mice, indicating a potential increase in the activity of mitochondria-associated membranes (MAMs), which act as critical hubs linking lipid droplet biogenesis, lipid metabolism, and cellular stress sensing. Given that DNASE1L3 is a secreted endonuclease whose synthesis and trafficking rely on the ER-Golgi-vesicular system,^23^ we hypothesized that its deficiency may impair endoplasmic reticulum (ER) homeostasis in KCs, thereby triggering ER stress-associated signaling pathways. Specifically, we proposed that DNASE1L3 loss activates unfolded protein response (UPR) pathways, thereby linking innate immune activation with metabolic reprogramming.

**Figure 4:**
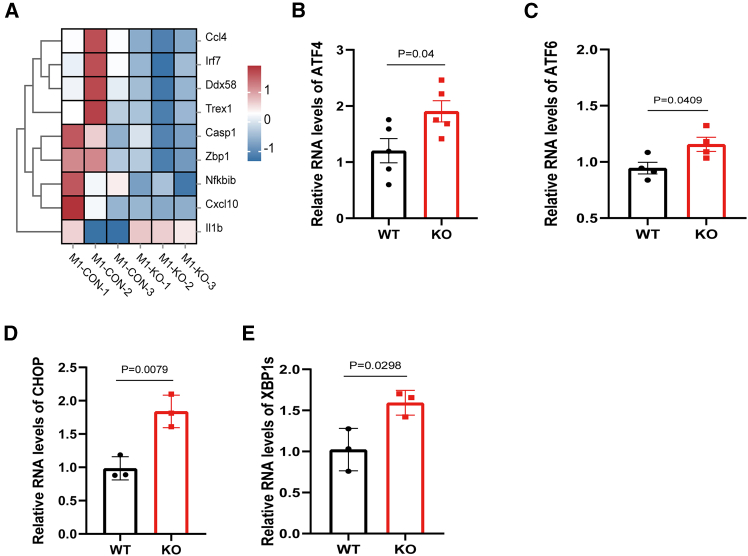
DNASE1L3 deficiency induces ER stress and activates canonical UPR signaling in KCs (A) Heatmap of differentially expressed genes in M1-polarized KCs (*n* = 3 per group), highlighting significant downregulation of DNA sensing and inflammation-associated genes in the KO group, including Ddx58, Trex1, Casp1, Nfkbib, and Il1b. (B–E) Quantitative RT-PCR analysis of key markers in ER stress and UPR pathways in M1-polarized KCs (*n* = 3–5 per group). (B) ATF4, a downstream effector of the PERK pathway, was significantly upregulated in the KO group (*p* = 0.0400). (C) ATF6 expression was also significantly increased (*p* = 0.0409). (D) Expression of CHOP, a pro-apoptotic ER stress marker, was markedly elevated in KO KCs (*p* = 0.0079). (E) The spliced isoform of XBP1 (XBP1s), indicative of IRE1 pathway activation, was significantly upregulated (*p* = 0.0298), collectively confirming activation of all three major UPR branches (PERK, ATF6, and IRE1). Data are presented as mean ± SEM. Statistical comparisons were performed using unpaired two-tailed Student’s *t* test. Exact *p* values are indicated; *p* < 0.05 was considered statistically significant.

To test this hypothesis, we analyzed RNA-seq profiles of KCs subjected to M1 polarization. Heatmap clustering revealed significant downregulation of cytosolic DNA sensors, including Zbp1, three prime repair exonuclease 1 (Trex1), DExD/H-box helicase 58 (Ddx58), and interferon regulatory factor 7 (Irf7), in KCs from *Dnase1l3*-deficient mice (Figure 4A).^24^^,^^25^^,^^26^^,^^27^ Although these genes are classical effectors of DAMP sensing and interferon signaling, their decreased expression may reflect a feedback suppression under chronic cfDNA exposure. Notably, these sensors also play roles in lipid droplet biogenesis, lipid metabolism, and inflammation, suggesting that their downregulation represents an adaptive but maladaptive shift, consistent with a state of immunometabolic imbalance in DNASE1L3-deficient KCs.

We next examined the activation status of canonical UPR pathways to evaluate ER functionality.^28^ Quantitative RT-PCR revealed significant upregulation of activating transcription factor 4 (Atf4, *p* = 0.0400; Figure 4B), a downstream effector of the PKR-like ER kinase (Perk) pathway, and activating transcription factor 6 (Atf6, *p* = 0.0409; Figure 4C), indicating activation of the ATF6 branch. Furthermore, expression levels of C/EBP homologous protein (Chop, *p* = 0.0079; Figure 4D) and the spliced isoform of X-box binding protein 1 (Xbp1, *p* = 0.0298; Figure 4E) were significantly increased, confirming activation of the inositol-requiring enzyme 1 (IRE1)-XBP1 pathway. Collectively, these results indicate simultaneous activation of all three major UPR branches in DNASE1L3-deficient KCs.

In summary, DNASE1L3 deficiency may be associated with the accumulation of DAMPs, which in turn could contribute to lipid metabolic dysregulation and ER stress in KCs. Coordinated activation of the PERK, ATF6, and IRE1-XBP1 branches of the UPR may sustain a positive feedback loop linking inflammation, cellular stress, and energy metabolism. This mechanistic axis may underlie the persistent proinflammatory activation and lipid accumulation in KCs, offering insights into the pathogenesis of fatty liver and related chronic liver diseases.

### DNASE1L3 deficiency induces redox imbalance and ferroptosis-associated metabolic reprogramming in the liver

**Figure 5:**
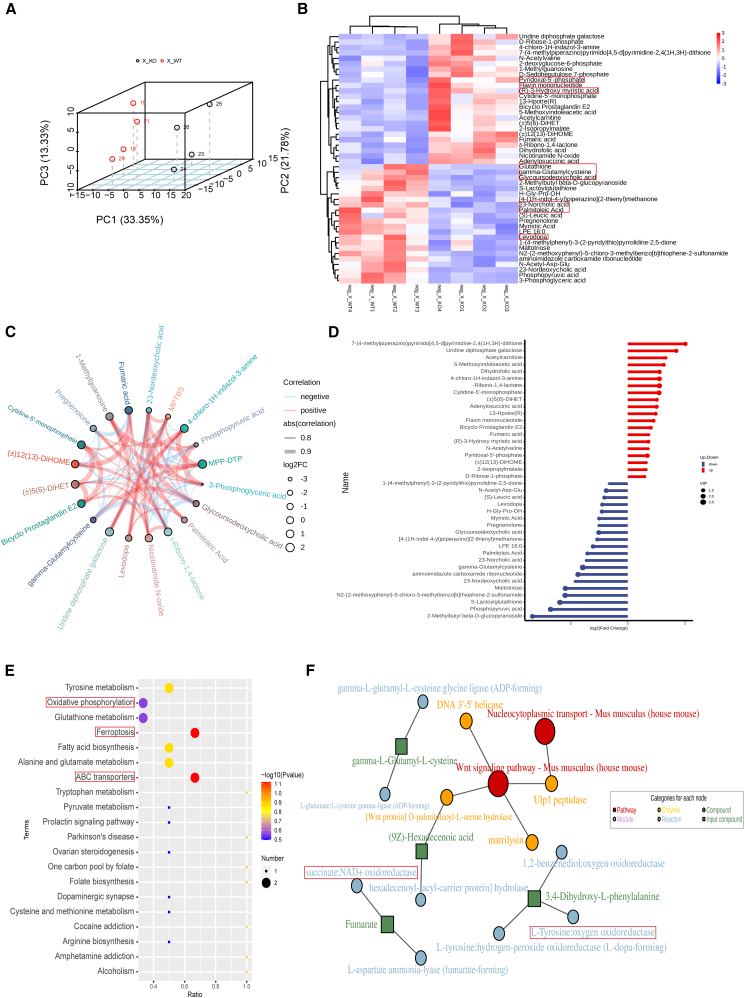
Metabolomic profiling reveals redox imbalance and ferroptosis-associated metabolic reprogramming in DNASE1L3-deficient livers (A) PCA 3D score plot demonstrating clear metabolic separation between WT and KO mice along PC1 (33.35% variance), PC2, and PC3, indicating substantial alterations in global hepatic metabolomes(*n* = 3 per group). (B) Hierarchical clustering heatmap of the top 50 differentially abundant metabolites between WT and KO groups. Relative abundance is color-coded from high (red) to low (blue). (C) Pathway-metabolite correlation network showing associations between enriched metabolic pathways and representative metabolites. Edge thickness corresponds to the absolute correlation coefficient; edge color indicates directionality (positive or negative), and node size reflects log_2_ fold change. (D) KEGG pathway enrichment analysis of differentially expressed metabolites. Bar plot illustrates the top 30 enriched pathways; red bars represent upregulated, and blue bars downregulated pathways. The *x* axis denotes –log_10_ (*p* value), and dot size indicates the number of mapped metabolites. (E) GSEA-based dot plot highlighting the negative enrichment of ferroptosis, oxidative phosphorylation, glutathione metabolism, and other redox-associated pathways in KO livers. (F) Topological analysis of the metabolic network reveals glutathione metabolism, nucleocytoplasmic transport, and Wnt signaling as central regulatory hubs in DNASE1L3-deficient mice. Node color reflects functional classification (red: pathway; orange: enzyme; green: metabolite input), and node size indicates topological centrality. Differential metabolites were identified based on variable importance in projection (VIP) > 1 and *p* < 0.05. Pathway enrichment and network analyses were performed using MetaboAnalyst and KEGG databases; pathways with –log_10_(*P*) > 1 were considered significantly enriched. Data are presented as mean ± SEM. Intergroup comparisons were conducted using two-tailed unpaired Student’s *t* test.

To comprehensively investigate the metabolic consequences of DNASE1L3 deficiency in the liver, we confirmed by western blot that DNASE1L3 expression was highest in the liver among metabolic organs (Figure S2B). We then performed untargeted metabolomic profiling in negative ion mode using liver tissues from 8-week-old WT and KO mice. Principal component analysis (PCA) revealed clear group separation along PC1 (33.35%), PC2 (21.78%), and PC3 (13.33%), indicating widespread metabolic alterations (Figure 5A).

Hierarchical clustering heatmap analysis (Figure 5B) revealed widespread dysregulation of key metabolites in KO livers. Dysregulated species included intermediates of fatty acid metabolism (e.g., palmitoleic acid and myristic acid), glutathione biosynthesis (e.g., γ-glutamylcysteine and glutathione), aromatic amino acid derivatives (e.g., L-DOPA and pyridoxal-5′-phosphate), and bile acid-related compounds (e.g., 23-norcholic acid and glycoursodeoxycholic acid). Volcano plots and pathway-metabolite correlation networks (Figures 5C and 5D) revealed that upregulated metabolites were primarily linked to inflammatory or oxidative pathways (e.g., (±)12(13)-DiHOME and fumaric acid), whereas downregulated metabolites were enriched in glutathione metabolism, bile acid synthesis, and central energy metabolism.

KEGG enrichment analysis (Figure 5E) further revealed significant accumulation of differential metabolites in pathways related to fatty acid biosynthesis, glutathione metabolism, tyrosine metabolism, and tryptophan metabolism. Notably, pathways associated with ferroptosis, oxidative phosphorylation, and ABC transporters were also enriched, implicating mitochondrial dysfunction and disrupted iron homeostasis as potential triggers of iron-dependent cell death. Metabolic network topology mapping (Figure 5F) identified key regulatory nodes including glutathione synthesis, Wnt signaling, and nucleocytoplasmic transport, where multiple enzymes (e.g., succinate: NAD^+^ oxidoreductase, L-tyrosine:oxygen oxidoreductase) were functionally interconnected within core energy metabolism circuits.

To validate the metabolomic findings, we assessed hepatic reactive oxygen species (ROS) levels by flow cytometry. KO livers exhibited significantly elevated ROS levels (*p* = 0.0046; Figure S2C), confirming the presence of oxidative stress and a predisposition to ferroptosis in the absence of DNASE1L3. Consistently, immunohistochemistry revealed decreased expression of the ferroptosis-suppressing enzyme glutathione peroxidase 4 (GPX4) and increased accumulation of the lipid peroxidation marker 4-hydroxynonenal (4-HNE) in KO livers compared with WT controls (Figures S2D–S2E).^29^^,^^30^

In summary, DNASE1L3 deficiency disrupts hepatic redox homeostasis, glutathione metabolism, and iron regulation, while concurrently activating proinflammatory signaling. These alterations converge on ferroptosis, forming a pathological immune-stress-metabolism axis that may drive the initiation and progression of fatty liver disease in DNASE1L3-deficient mice.

### Proteomic analysis reveals hepatic metabolic reprogramming and ferroptosis pathway activation in DNASE1L3-deficient mice

To validate our transcriptomic and metabolomic findings at the protein level, we performed tandem mass tag (TMT)-based quantitative proteomic profiling of liver tissues from KO and WT mice. Subcellular localization analysis of differentially expressed proteins (DEPs) revealed broad distribution across major organelles, including the cytoplasm (25.88%), nucleus (20.00%), ER (10.59%), mitochondria, microsomes, peroxisomes, and the Golgi apparatus (Figure S2F), suggesting global organellar dysfunction resulting from DNASE1L3 deficiency.

GO enrichment analysis revealed that DEPs were significantly associated with aromatic amino acid metabolism, lipid synthesis and catabolism, iron ion homeostasis, and redox balance (Figure S2G). Enriched cellular components were mainly associated with the ER and mitochondria, and functional terms included iron ion binding, peroxidase activity, and nuclease activity, indicating potential dysregulation of oxidative and ferroptotic pathways.

**Figure 6:**
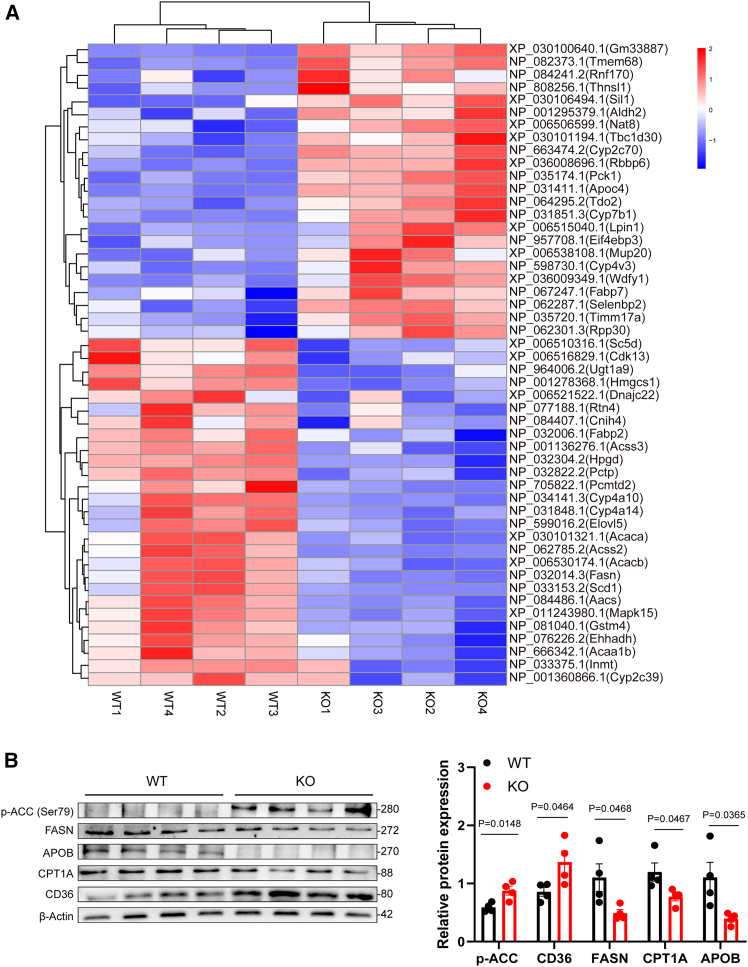
Proteomic analysis reveals subcellular dysfunction and metabolic disruption induced by DNASE1L3 deficiency (A) Heatmap showing hierarchical clustering of DEPs in WT and KO liver samples (*n* = 4 per group). Color gradient represents relative protein abundance (red: high; blue: low). Proteins were functionally annotated as transporters, metabolic enzymes, or structural regulators involved in lipid metabolism, oxidative stress, and organelle function. (B) Western blot analysis of key lipid metabolism-related proteins, including *p*-ACC (Ser79), FASN, APOB, CPT1A, and CD36, in liver tissues from WT and KO mice. Quantification revealed significant upregulation of *p*-ACC and CD36, and downregulation of FASN, CPT1A, and APOB in KO livers (*p* < 0.05 for all comparisons; *n* = 4 per group). Data are presented as mean ± SEM. Statistical comparisons were performed using unpaired two-tailed Student’s *t* test. Exact *p* values are indicated; *p* < 0.05 was considered statistically significant. DEPs were defined by fold change >1.5 and *p* < 0.05.

Hierarchical clustering analysis revealed distinct proteomic profiles between KO and WT groups (Figure 6A). Within the lipid metabolism pathway, core enzymes involved in fatty acid synthesis—fatty acid synthase (FASN), acetyl-CoA carboxylase 1 (ACC1), and stearoyl-CoA desaturase 1 (SCD1)—as well as their upstream substrate-producing enzymes—acyl-CoA synthetase short-chain family member 2 (ACSS2), ACSS3, and acetoacetyl-CoA synthetase (AACS)—were significantly downregulated, whereas the β-oxidation enzyme acyl-CoA oxidase 1 (ACOX1) was increased. Enoyl-CoA hydratase/3-hydroxyacyl-CoA dehydrogenase (EHHADH) and acetyl-CoA acyltransferase 1B (ACAA1B) were decreased, suggesting disturbed fatty acid catabolism.

To confirm these proteomic patterns, we performed western blot analysis (Figure 6B). The results verified decreased expression of FASN, CPT1A, and APOB, accompanied by increased phospho-ACC (Ser79) and CD36, consistent with the proteomic dataset.^31^^,^^32^^,^^33^ These data indicate complex alterations in hepatic lipid metabolism under DNASE1L3 deficiency, including enhanced lipid uptake, suppressed *de novo* lipogenesis, and impaired fatty acid oxidation and export.

Proteomic signatures also pointed to ER stress. The UPR sensor endoplasmic reticulum to nucleus signaling 1 (ERN1/IRE1α) was significantly upregulated, whereas chaperones involved in protein folding and redox buffering, such as glutathione S-transferase mu 4 (GSTM4) and DnaJ heat shock protein family member C22 (DNAJC22), were downregulated. Upregulation of ER-associated degradation (ERAD) components—including SIL1 nucleotide exchange factor (SIL1), retinoblastoma-binding protein 6 (RBBP6), and ring finger protein 170 (RNF170)—indicated a compensatory response to misfolded protein accumulation. Moreover, reduced expression of Reticulon 4 (RTN4), coupled with increased expression of translocase of inner mitochondrial membrane 17A (TIMM17A) and TBC1 domain family member 30 (TBC1D30), suggested disrupted ER-mitochondria-Golgi vesicular trafficking.

Redox imbalance was also evident in KO livers. Key NADPH-generating enzymes—including malic enzyme 1 (ME1), ATP citrate lyase (ACLY), and UDP-glucuronosyltransferase family 1 member A9 (UGT1A9)—were downregulated, consistent with impaired antioxidant capacity. Conversely, upregulation of aldehyde dehydrogenase 2 (ALDH2) and phosphoenolpyruvate carboxykinase 1 (PCK1) may represent compensatory efforts to mitigate lipid peroxidation and maintain redox homeostasis.

Critically, several ferroptosis-related proteins were differentially regulated. The pro-ferroptotic enzyme acyl-CoA synthetase long-chain family member 4 (ACSL4) was upregulated, whereas GPX4, a central anti-ferroptotic enzyme, was downregulated. Iron storage proteins ferritin heavy chain 1 (FTH1) and ferritin light chain (FTL) were elevated, indicative of increased hepatic iron accumulation. Upregulation of selenium-binding protein 2 (SELENBP2) and downregulation of thiosulfate sulfurtransferase-like domain containing 1 (TSTD1) and nudix hydrolase 9 (NUDT9) further supported impaired mitochondrial and antioxidant defenses in DNASE1L3-deficient livers.

Collectively, these proteomic data suggest that DNASE1L3 deficiency may contribute to hepatic metabolic dysregulation through impaired lipid metabolism, ER stress induction, compromised antioxidant defenses, and altered iron homeostasis, ultimately converging on ferroptosis activation. These findings are consistent with transcriptomic and metabolomic profiles and support a potential mechanistic cascade involving lipid metabolic reprogramming, ER stress, oxidative stress, and ferroptosis, which may contribute to fatty liver pathogenesis in DNASE1L3-deficient mice.

### DNASE1L3 deficiency induces extensive genome-wide mutations and chromosome 4-specific mutation clustering in mice

In the preceding analyses, we demonstrated that DNASE1L3 deficiency profoundly disrupts hepatic immune homeostasis, metabolic balance, and ferroptosis regulation. Given that DNASE1L3 is a nuclease possessing a nuclear localization sequence (NLS),^23^ its absence may impair the clearance of endogenous DNA, thereby promoting genomic instability. To test this hypothesis, we conducted whole-genome resequencing on tail DNA samples from KO mice. Detected variants were compared against publicly available mouse variant databases (e.g., Mouse Genome Project and dbSNP), and only shared variants absent from these reference datasets were retained for downstream analysis to evaluate DNASE1L3-related genomic alterations.

**Figure 7:**
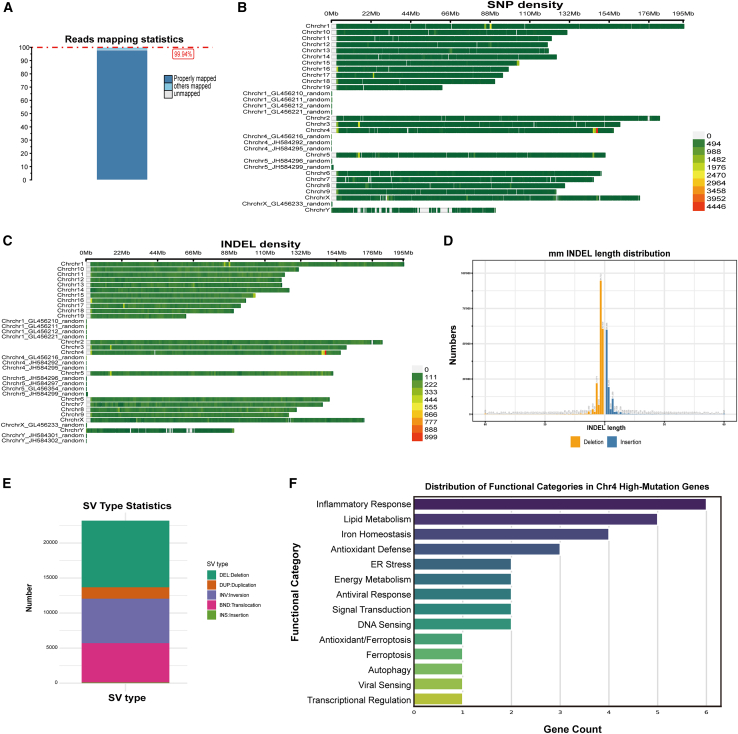
Whole-genome resequencing reveals widespread genomic alterations and chromosome 4-specific mutation enrichment in DNASE1L3-deficient mice (A) Read mapping statistics showing a high overall mapping rate (99.94%) based on alignment to the mouse reference genome using BWA-MEM2 (*n* = 3 per group). (B) SNP density distribution across chromosomes and genomic intervals. Mutation hotspots were prominently detected on chromosome 4. (C) INDEL density distribution across chromosomes. Chr4 exhibits regional INDEL clustering, suggesting cumulative structural variation. (D) Length distribution of detected INDELs, showing a predominance of short (1–5 bp) events, with a minority of longer insertions/deletions (>50 bp). (E) Summary of structural variant (SV) types identified in DNASE1L3-deficient mice, including deletions (DEL), duplications (DUP), inversions (INV), translocations (BND), and insertions (INS), with deletions and insertions being the most prevalent. (F) Functional categorization of Chr4 high-mutation genes reveals enrichment in pathways related to inflammation, lipid metabolism, oxidative stress, iron homeostasis, and ferroptosis.

Sequencing data aligned to the mm10 reference genome using BWA-MEM2 achieved an average mapping rate of 99.94%, with the majority of reads properly paired (Figure 7A), indicating high data quality. Across the genome, approximately 2.25 million single nucleotide polymorphisms (SNPs) and 380,000 insertions/deletions (INDELs) were identified. These variants were distributed unevenly across chromosomes, with chromosome 4 (Chr4) exhibiting notably high mutation density and distinct clustering (Figures 7B and 7C), suggesting it may act as a mutation hotspot in the absence of DNASE1L3.

INDEL length analysis showed a predominance of short variants (1–5 bp), along with a subset of medium-to-long insertions or deletions (>50 bp) (Figure 7D), which may disrupt coding sequences or regulatory regions. Structural variant (SV) analysis revealed widespread genomic rearrangements, including deletions (DEL), duplications (DUP), inversions (INV), translocations (BND), and insertions (INS), with deletions and insertions being the most frequent, exceeding 15,000 and 10,000 events, respectively (Figure 7E). These findings suggest that DNASE1L3 deficiency promotes both point mutations and large-scale genomic remodeling.

Annotation of the high-density mutation regions on Chr4 identified 33 protein-coding genes harboring potential functional variants. Functional enrichment analysis indicated involvement of these genes in lipid metabolism, iron homeostasis, oxidative stress response, ER stress, and ferroptosis regulation. Additionally, mutations were detected in genes linked to inflammation and nucleic acid sensing (details are provided in Table S2; Figure 7F), supporting a role for these variants in immunometabolic dysregulation.

In summary, DNASE1L3 deficiency may be associated with widespread genomic mutations, with Chr4 exhibiting a notable accumulation of mutational events affecting genes related to immune regulation, metabolic homeostasis, and cellular stress responses. These alterations may contribute to intrahepatic immune–metabolic remodeling under chronic inflammatory conditions and support a potential role for DNASE1L3 in maintaining genomic and hepatic stability.

## Discussion

By integrating immunophenotyping, transcriptomics, proteomics, metabolomics, and histological analyses, our study highlights DNASE1L3 as a potential regulator of hepatic immune-metabolic homeostasis. DNASE1L3 deficiency perturbed myeloid differentiation and promoted proinflammatory polarization of KCs, accompanied by enrichment of DAMP sensing pathways. Although cfDNA was not directly quantified, DNASE1L3 is a well-established extracellular endonuclease for nucleic acid clearance.^34^^,^^35^ Its absence may facilitate cfDNA accumulation, sustaining PRR activation and amplifying inflammatory responses.

The activation of DAMP-PRR signaling in DNASE1L3-deficient livers was associated with lipid metabolic reprogramming, ER stress, and ferroptotic susceptibility. These results suggest that immune activation, metabolic disruption, and cell death pathways collectively contribute to fatty liver progression. In contrast to previous studies that mainly emphasized hepatocyte-intrinsic mechanisms such as lipid dysregulation, mitochondrial dysfunction, or ROS accumulation involving sterol regulatory element-binding protein 1c (SREBP1c) and peroxisome proliferator-activated receptor gamma (PPARγ) pathways,^36^^,^^37^^,^^38^^,^^39^ our data highlight innate immune regulation as a potential initiating factor. We therefore propose a cfDNA–PRR–KC axis that may underlie an immune–metabolic subtype of NAFLD, providing a possible explanation for disease heterogeneity and expanding the functional scope of DNASE1L3 beyond classical immune contexts.^40^

Notably, although proteomic profiling revealed a global downregulation of lipogenic enzymes, subsequent protein validation demonstrated that DNASE1L3 deficiency does not simply enhance fatty acid synthesis but instead induces a compensatory, multilayered metabolic reprogramming. Phosphorylation of ACC at Ser79 leads to its inactivation, thereby suppressing *de novo* lipogenesis. Meanwhile, upregulation of CD36 facilitates lipid uptake, whereas the downregulation of CPT1A and APOB indicates impaired fatty acid oxidation and VLDL secretion, resulting in an “input > output” lipid flux imbalance.^41^^,^^42^ Mechanistically, DNASE1L3 deficiency activates all three canonical branches of the UPR (PERK, IRE1, and ATF6), disrupting ER homeostasis in hepatic macrophages, promoting M1 polarization and proinflammatory activation, and exacerbating hepatic oxidative stress and ferroptotic susceptibility.^43^^,^^44^ These interdependent processes collectively explain the observed temporal pattern in which KC activation and inflammatory remodeling appear as early as 12 weeks, whereas overt lipid droplet accumulation becomes evident only after prolonged stress exposure. This model is further supported by our whole-genome resequencing data showing chromosome 4-enriched mutations in genes related to lipid metabolism, oxidative stress, iron homeostasis, and inflammation. Such genomic instability suggests that defective DNA degradation caused by DNASE1L3 loss may concurrently promote cfDNA-driven immune activation and compromise genome integrity, thereby perpetuating chronic immunometabolic dysregulation and progressive hepatic steatosis.

Despite the mechanistic depth and comprehensive multi-omics approach, several limitations should be acknowledged. First, the absence of a KC-specific DNASE1L3 KO model limits definitive attribution of the observed phenotypes to cell-intrinsic effects. Second, although ferroptosis was implicated by multi-omics signatures and supported by immunohistochemical detection of ferroptosis-associated proteins such as GPX4 and 4-HNE, ultrastructural confirmation (e.g., transmission electron microscopy) and functional rescue experiments using ferroptosis inhibitors such as GPX4 agonists were not performed. Third, while cfDNA appears to function as a central driver of sterile inflammation, direct evidence through DNASE1L3 supplementation or PRR inhibition is warranted to establish causality. These limitations highlight important avenues for future research.

In conclusion, our study supports DNASE1L3 as a key regulator of hepatic immune-metabolic homeostasis. Its deficiency contributes to chronic innate immune activation, metabolic reprogramming, ER stress, ferroptosis, and genomic instability, ultimately promoting NAFLD progression. These findings broaden the potential functional scope of DNASE1L3 beyond its canonical nuclease activity and suggest that targeting cfDNA-PRR signaling, ER stress, ferroptotic pathways, or genomic stability may provide therapeutic opportunities for immune-metabolic subtypes of NAFLD.

### Limitations of the study

While our study provides a comprehensive multi-omics characterization of DNASE1L3-deficient mice, several limitations should be noted. First, the data were obtained from global KO animals, which cannot fully exclude potential systemic or developmental effects beyond the liver microenvironment. Conditional or tissue-specific deletion models would help clarify the cell-autonomous role of DNASE1L3. Second, although proteomic and transcriptomic analyses suggest mechanistic links among cfDNA accumulation, KC activation, ER stress, and ferroptosis, direct causal relationships require further validation through genetic or pharmacologic intervention. Third, our study focused on male mice to reduce sex-related variability; the potential influence of sex on DNASE1L3-associated metabolic and immune phenotypes remains to be investigated. Finally, while integration of multiple omics layers improves robustness, cross-platform differences and temporal heterogeneity may introduce bias, underscoring the need for future longitudinal and single-cell studies.

## Resource availability

### Lead contact

Further information and requests for resources and reagents should be directed to and will be fulfilled by the lead contact, Prof. Quanyan Liu (liuqy@tmu.edu.cn).

### Materials availability

This study did not generate new unique reagents. DNASE1L3-deficient mice on a C57BL/6 background were generated by Cyagen Biosciences (contract no. KOAI190426JW1).

### Data and code availability

• The RNA-seq data generated in this study have been deposited in the NCBI Sequence Read Archive (SRA) under the BioProject accession number PRJNA1332920, with individual submissions SUB15658739, SUB15659780, and SUB15666046.
• The TMT-based quantitative proteomic dataset has been deposited to the ProteomeXchange Consortium via the PRIDE partner repository under the accession number PXD068786 and is publicly accessible at: https://www.ebi.ac.uk/pride/archive/projects/PXD068786.
• The untargeted metabolomics dataset has been deposited in MetaboLights under the accession number MTBLS13057, titled multi-omics analysis reveals immune-metabolic dysregulation in DNASE1L3-deficient mouse liver.
• The whole-genome resequencing data are included in the same BioProject PRJNA1332920.
• This study did not generate any original code.
• All software used in this study is listed in the key resources table with corresponding references.

## Acknowledgments

This work was supported by grants from the 10.13039/100014718National Natural Science Foundation of China (nos. 82171563, 82372902, and 82403370). The authors thank the Institute of General Surgery, 10.13039/501100010104Tianjin Medical University, for providing experimental support and research infrastructure. Additional appreciation is extended to colleagues who supported flow cytometry analysis, imaging experiments, animal care, and statistical consultation during manuscript preparation.

## Author contributions

L.L., J.Y., B.Z., and P.L., contributed equally to this work and share co-first authorship; L.L. and Q.L., conceptualized and supervised the study; L.L., performed most of the experiments and data analysis; J.Y. and B.Z., contributed to flow cytometry and animal model establishment; P.L., carried out transcriptomic and proteomic analyses; Z.M. and Y.S., assisted in metabolomic profiling and bioinformatics interpretation; J.L. and B.L., contributed to histological analysis and immunofluorescence; L.L. and Q.L., wrote the manuscript with input from all authors. All authors have read and agreed to the published version of the manuscript.

## Declaration of interests

The authors declare no competing interests.

## STAR★Methods

### Key resources table

**Table:** 

REAGENT or RESOURCE	SOURCE	IDENTIFIER
**Antibodies**		

FITC anti-mouse Lineage Cocktail with Isotype Ctrl	BioLegend	Cat#133301; RRID: AB_2562613
PE/Cy7 anti-mouse CD34 (Clone RAM34)	BioLegend	Cat#119325; RRID: AB_756085
PE anti-mouse Ly-6A/E (Clone D7)	BioLegend	Cat#108107; RRID: AB_313344
APC anti-mouse CD117 (Clone 2B8)	BioLegend	Cat#105811; RRID: AB_313221
PerCP/Cy5.5 anti-mouse CD16/32 (Clone 93)	BioLegend	Cat#156623; RRID: AB_2721485
FITC anti-mouse F4/80 (Clone BM8)	BioLegend	Cat#123107; RRID: AB_893481
APC anti-mouse CD86 (Clone GL1)	BioLegend	Cat#105113; RRID: AB_2075033
APC anti-mouse Gr-1 (Clone RB6-8C5)	BioLegend	Cat#108408; RRID: AB_313376
PE anti-mouse/human CD11b (Clone M1/70)	BioLegend	Cat#101207; RRID: AB_312791
FITC anti-mouse CD206 (Clone C068C2)	BioLegend	Cat#141707; RRID: AB_10960185
PerCP/Cy5.5 anti-mouse Ly-6C (Clone HK1.4)	BioLegend	Cat#128005; RRID: AB_1659243
FITC anti-mouse CD3 (Clone 17A2)	BioLegend	Cat#981002; RRID: AB_2561463
PE anti-mouse CD4 (Clone GK1.5)	BioLegend	Cat#100407; RRID: AB_312691
PerCP/Cy5.5 anti-mouse CD8a (Clone 53–6.7)	BioLegend	Cat#100733; RRID: AB_312745
Anti-TNF (IHC, rabbit polyclonal, 1:200)	Servicebio	Cat#GB11188-100
Anti-IL-6 (IHC, rabbit polyclonal, 1:200)	Servicebio	Cat#GB11117-100
Anti-MCP-1 (IHC, rabbit polyclonal, 1:200)	Servicebio	Cat#GB11199-100
Anti-F4/80 (IHC, rabbit polyclonal, 1:200)	Servicebio	Cat#GB11027-100
Anti-HMGB1 (IHC, rabbit polyclonal, 1:200)	Servicebio	Cat#GB11103-100
Anti-GPX4 (IHC, rabbit polyclonal, 1:200)	Abclonal	Cat# A11243
Anti-4-HNE (IHC, rabbit polyclonal, 1:200)	Servicebio	Cat#GB150073-100
Anti-DNASE1L3 (WB, rabbit polyclonal, 1:1000)	Genetex	Cat#GTX114363
Anti–phospho-ACC1 (Ser79) (WB, rabbit polyclonal, 1:1000)	ABclonal	Cat# AP0298; RRID: AB_2861348
Anti-APOB (WB, rabbit monoclonal, 1:1000)	ABclonal	Cat# A4184; RRID: AB_2861339
Anti-CD36 (WB, rabbit polyclonal, 1:1000)	ABclonal	Cat# A5792; RRID: AB_2861375
Anti-CPT1A (WB, rabbit monoclonal, 1:1000)	ABclonal	Cat# A27657; RRID: AB_2861327
Anti-FASN (WB, mouse monoclonal, 1:1000)	Proteintech	Cat# 66591-1-Ig; RRID: AB_2880791
Anti–β-Actin (WB, mouse monoclonal, 1:5000)	Proteintech	Cat# 66009-1-Ig; RRID: AB_2687938
HRP-conjugated secondary antibody	Servicebio	Cat#G1210; RRID:N/A

**Biological samples**		

Liver tissues from C57BL/6J mice	This paper	N/A

**Chemicals, peptides, and recombinant proteins**		

Recombinant mouse IL-4	Novoprotein	Cat#CK74
Lipopolysaccharide (LPS)	Sigma-Aldrich	Cat#L6529
Collagenase IV	Solarbio	Cat#C8160
DNase I	Solarbio	Cat#D8072
Oil Red O Staining Kit	Servicebio	Cat#G1015-100 ML
Lipid Fixative	Servicebio	Cat#G1119-100 ML
Hematoxylin	Servicebio	Cat#G1004
TRIzol reagent	Invitrogen	Cat#15596026
ABScript III RT Master Mix	Abclonal	Cat#RK20429
PerfectStart Green qPCR SuperMix	TransGen Biotech	Cat#AQ601
NEBNext Ultra II RNA Library Prep Kit	NEB	Cat#E7770
TMT10plex Isobaric Label Reagent Set	Thermo Fisher	Cat#90110
Thioglycolate broth	Sigma-Aldrich	Cat#T9032

**Deposited data**		

Proteomics data	This paper	ProteomeXchange: PXD068786
Metabolomics data	This paper	MetaboLights: MTBLS13057
Whole-genome resequencing data	This paper	NCBI SRA: PRJNA1332920
RNA-seq data	This paper	NCBI SRA: PRJNA1332920 (Submissions: SUB15658739, SUB15659780, SUB15666046)

**Experimental models: Organisms/strains**		

Dnase1l3 knockout mice (C57BL/6 background)	Cyagen Biosciences	Contract No. KOAI190426JW1; RRID:N/A
C57BL/6J mice	Beijing Vital River Laboratory Animal Technology	RRID:IMSR_JAX:000664

**Oligonucleotides**		

gRNA sequences for Dnase1l3	This paper	Table S1
Genotyping primers (WT/KO)	This paper	Table S1
qPCR primers (Atf4, Atf6, Chop, Xbp1s, Tlr4, Nlrp3, Zbp1)	This paper	Table S1

**Software and algorithms**		

FlowJo v10.8.0	BD Biosciences	https://www.flowjo.com/
FACSDiva v9.0	BD Biosciences	Installed with cytometer
GraphPad Prism v9.0	GraphPad Software	https://www.graphpad.com/
MaxQuant v1.6.17.0	Cox Lab	https://www.maxquant.org/
DESeq2 v1.30.1	Bioconductor	https://bioconductor.org/packages/DESeq2/
fastp v0.23.2	OpenGene	https://github.com/OpenGene/fastp
BWA-MEM2 v2.2.1	Heng Li Lab	https://github.com/bwa-mem2
GATK v4.1	Broad Institute	https://gatk.broadinstitute.org/
Manta v1.6.0	Illumina	https://github.com/Illumina/manta
SnpEff v5.1	Cingolani Lab	http://snpeff.sourceforge.net/
Metascape (2019 release)	Zhou Lab	https://metascape.org/
ImageJ/Fiji v1.53	NIH	https://imagej.net/software/fiji/
BMKCloud	Biomarker Technologies	http://www.biocloud.net
HISAT2 v2.2.1	Kim Lab	http://daehwankimlab.github.io/hisat2/

### Experimental model and study participant details

#### Mice

**Species/strain and genotype:** Wild-type (WT) C57BL/6J mice and Dnase1L3 knockout (Dnase1l3^−/−^, KO) mice on a C57BL/6 background were used for all experiments unless otherwise stated. Dnase1l3^−/−^ mice were generated by Cyagen Biosciences using CRISPR/Cas9-mediated gene deletion.

**Age and sex:** Male mice aged 6–8 weeks (for initial experiments) or the age indicated in each figure legend (8–50 weeks for phenotyping) were used. Only male mice were included to avoid hormonal cycle–associated variability. This design choice prevents the assessment of sex-specific biological effects.

**Housing and care:** All animals were housed in specific pathogen–free (SPF) facilities at the Laboratory Animal Center of Tianjin Medical University under controlled conditions (temperature 21°C–23°C; humidity 40–60%; 12-h light/dark cycle). Animals were experimentally naive, had not undergone previous procedures, and had *ad libitum* access to food and water.

**Ethical approval:** All animal procedures were reviewed and approved by the Institutional Animal Care and Use Committee (IACUC) of Tianjin Medical University General Hospital (Approval No. IRB2021-DWFL-103) and were performed in accordance with institutional, national, and international guidelines for the care and use of laboratory animals.

#### Primary cells

**Kupffer cells:** Kupffer cells were isolated from male C57BL/6 WT and Dnase1l3^−/−^ mice by liver perfusion, enzymatic digestion, Percoll gradient centrifugation, and differential adhesion as described in the STAR Methods. Cells were used immediately after isolation and were not maintained in long-term culture.

**Peritoneal macrophages:** Peritoneal macrophages were collected from male C57BL/6 mice via peritoneal lavage under steady-state conditions or following thioglycolate elicitation. Cells were enriched by adherence in RPMI-1640 medium supplemented with 10% FBS and 1% penicillin–streptomycin. All primary cell isolations were conducted following the same IACUC-approved protocols listed above.

#### Cell lines

This study did not include immortalized or transformed cell lines.

#### Human participants

This study did not involve human participants.

### Method details

#### High-fat diet (HFD) feeding model

Wild-type and DNASE1L3-deficient mice were randomly assigned to either a normal chow diet (NCD, 10% kcal from fat) or a high-fat diet (HFD, 60% kcal from fat; D12492, Research Diets Inc.). Mice were fed *ad libitum* for 16 weeks, with body weight monitored weekly. At the endpoint, mice were fasted overnight before sacrifice and tissue collection.

#### Isolation of Kupffer cells

Following anesthesia, hepatic portal perfusion was performed with PBS to remove circulating blood. Livers were minced and digested in Hank’s balanced salt solution containing 0.05% collagenase IV (Solarbio, Cat#C8160) and 0.002% DNase I (Solarbio, Cat#D8072) at 37°C for 30 min with shaking. Digested tissue was filtered through a 200-mesh nylon strainer. Hepatocytes were removed by centrifugation at 800 rpm for 5 min, and the supernatant was centrifuged at 300 × g for 10 min to collect non-parenchymal cells. The pellet was resuspended in RPMI 1640 medium (10% FBS, 1% penicillin–streptomycin) and plated into uncoated 6-well plates. After 2 h at 37°C with 5% CO_2_, adherent Kupffer cells were retained.

#### Kupffer cell polarization assay

Kupffer cells were cultured in complete RPMI 1640 medium for 24 h, followed by stimulation with 100 ng/mL lipopolysaccharide (LPS; Sigma-Aldrich, Cat#L6529) for M1 polarization or 10 ng/mL recombinant mouse IL-4 (Novoprotein, Cat#CK74) for M2 polarization for 24 h.

#### Peritoneal macrophage isolation

Resident peritoneal macrophages were isolated by lavage with 5 mL sterile PBS containing 3% FBS. For thioglycolate-elicited macrophages, 2 mL of 3% thioglycolate broth (Sigma-Aldrich, Cat#T9032) was injected intraperitoneally, and cells were harvested 72 h later by lavage. Cells were centrifuged at 300 × g for 5 min, resuspended in RPMI 1640 medium, and plated. After 2 h, non-adherent cells were removed by washing with PBS, and adherent cells were retained.

#### Flow cytometry

Liver tissues were enzymatically digested and mechanically dissociated to generate single-cell suspensions. Red blood cells were lysed using ACK buffer. Fc receptors were blocked with anti-mouse CD16/CD32 antibody (clone 2.4G2; BioLegend, Cat#101319). Cells were then stained with fluorophore-conjugated antibodies (see key resources table) at 4°C for 15 min. Samples were acquired on a BD FACSCanto II cytometer with FACSDiva software (v9.0) and analyzed in FlowJo (v10.8). Data acquisition and gating were performed blinded to group allocation.

#### Immune subsets were defined as follows

• Kupffer cells: F4/80^+^ CD11bˆint
• CMPs: Lin^−^ Sca-1^-^ c-Kit^+^ CD34^+^ CD16/32^-^
• GMPs: Lin^−^ Sca-1^-^ c-Kit^+^ CD34^+^ CD16/32^+^
• MEPs: Lin^−^ Sca-1^-^ c-Kit^+^ CD34^−^ CD16/32^-^
• CD8^+^ T cells: CD3^+^ CD8^+^
• CD4^+^ T cells: CD3^+^ CD4^+^
• Classical monocytes (C-Mo): CD11b^+^ GR1^-^ Ly6Cˆhigh
• Non-classical monocytes (NC-Mo): CD11b^+^ GR1^-^ Ly6C^−^
• Neutrophils: CD11b^+^ Ly6Cˆint GR1^+^
• M1-like macrophages: F4/80^+^ CD11b^+^ CD86^+^
• M2-like macrophages: F4/80^+^ CD11b^+^ CD206^+^

#### Immunohistochemistry (IHC)

Liver tissues were fixed in 4% paraformaldehyde overnight, embedded in paraffin, and sectioned at 4 μm. After deparaffinization and rehydration, antigen retrieval was performed in citrate buffer (pH 6.0) at ∼95°C for 15 min. Endogenous peroxidase was quenched with 3% H_2_O_2_, and nonspecific binding blocked with 5% bovine serum albumin (BSA). Sections were incubated overnight at 4°C with primary antibodies against TNF, IL-6, MCP-1, F4/80, HMGB1, GPX4, and 4-HNE (all from Servicebio; details in the key resources table), followed by HRP-conjugated secondary antibody (Servicebio, Cat#G1210, 1:500 dilution). Signals were developed with DAB, counterstained with hematoxylin, dehydrated, and mounted.

#### Oil Red O staining

Liver tissues were fixed in lipid-specific fixative (Servicebio, Cat#G1119-100 ML), cryosectioned at 8 μm, stained with Oil Red O (Servicebio, Cat#G1015-100 ML) for 10 min, differentiated in 75% ethanol, counterstained with hematoxylin. After rinsing with distilled water and bluing, sections were mounted with glycerol gelatin and visualized under a light microscope.

#### Histological and ImageJ-based quantification

Oil Red O, and IHC-stained sections were imaged under a light microscope and quantified using ImageJ/Fiji.^45^ Lipid droplet area was measured using color deconvolution, expressed as % of total tissue area. IHC DAB intensity was quantified in ≥5 random fields per section, analyzed in a blinded manner.

#### Quantitative real-time PCR

Total RNA was extracted with TRIzol reagent (Invitrogen, Cat#15596026) and reverse-transcribed using ABScript III RT Master Mix (Abclonal, Cat#RK20429). qPCR was performed with PerfectStart Green qPCR SuperMix (TransGen Biotech, Cat#AQ601) on a CFX96 system (Bio-Rad). Primer sequences are listed in Table S1.

#### RNA sequencing

Total RNA was extracted and libraries prepared with NEBNext Ultra II RNA Library Prep Kit (NEB, Cat#E7770). Sequencing was performed on Illumina NovaSeq. Reads were aligned to the mouse reference genome (GRCm38) using HISAT2,^46^ and differential expression was analyzed with DESeq2.^47^ Genes with |log_2_FC| > 1 and FDR <0.05 were considered significant.

#### TMT-based proteomics

Proteins were extracted and enzymatically digested, followed by labeling with TMT reagents. The labeled peptides were analyzed by liquid chromatography–tandem mass spectrometry (LC-MS/MS). Protein identification and quantification were performed using MaxQuant^48^ software against the UniProt Mus musculus protein database. Differentially expressed proteins were defined as those with a fold change >1.5 and a *p*-value <0.05.

#### Untargeted metabolomics

Metabolites were extracted using a methanol–acetonitrile–water mixture (2:2:1, v/v/v) and analyzed by ultra-high-performance liquid chromatography–mass spectrometry (UHPLC-MS) in negative ion mode. Metabolic alterations were visualized using PCA, volcano plots, and KEGG pathway enrichment analysis. Differential metabolites were identified based on a variable importance in projection (VIP) score >1 and a *p*-value <0.05. Pathway enrichment was performed using Metascape.^49^

#### Whole-genome resequencing and variant annotation

Genomic DNA was extracted from tails, libraries prepared with TruSeq DNA PCR-Free Kit (Illumina), and sequenced on NovaSeq 6000 with paired-end 150 bp reads. Raw reads were quality-filtered using fastp(v0.23.2),^50^ and high-quality sequences were aligned to the mouse reference genome (GRCm39/mm39) using BWA-MEM2 (v2.2.1).^51^SNPs and INDELs were identified with GATK HaplotypeCaller.^52^ Structural variants were detected with Manta(v1.6.0)^53^ and annotated by SnpEff(v5.1).^54^ Mutation-enriched regions were analyzed with Metascape.^49^

### Quantification and statistical analysis

All data are presented as mean ± standard error of the mean (SEM) unless otherwise stated. Statistical details, including the exact *p* values, *n* values, and the tests used, are provided in the corresponding figure legends or directly annotated within figures.

• **Replication**: Each experiment was performed with at least three independent biological replicates. Animal experiments included ≥3 mice per group unless otherwise noted.
• **Randomization**: Mice were randomly assigned to experimental groups.
• **Blinding**: Histology, ImageJ quantification, and flow cytometry analysis were performed blinded to group allocation.
• **Inclusion/exclusion**: All animals were included unless they died from non-experimental causes; no data were excluded.
• **Sample size estimation**: Sample sizes were based on prior studies and standard practice in the field, not on formal power calculations.

Comparisons between two groups used unpaired two-tailed Student’s *t* test (parametric) or Mann–Whitney U test (non-parametric). For multiple groups, one-way ANOVA with Tukey’s post hoc test was applied. Normality of data distribution was assessed using the Shapiro–Wilk test. Analyses were performed in GraphPad Prism v9.0.

The value of n represents individual mice for *in vivo* studies or independent biological replicates for *in vitro* assays, as detailed in figure legends. A *p*-value <0.05 was considered statistically significant. Exact *p* values are indicated directly in the figures rather than using asterisks.
