# Supplementary material for: Systematic profiling reveals hepatic immune and metabolic dysregulation in DNASE1L3-deficient mice
Source: iScience. 2025 Nov 22;28(12):114198. doi: 10.1016/j.isci.2025.114198 (PMC12741396; doi:10.1016/j.isci.2025.114198)
Supplement: Document S1. Figures S1 and S2 and Tables S1 and S2 [file mmc1.pdf]

iScience, Volume *28*

Supplemental information

Systematic profiling reveals hepatic immune and metabolic dysregulation in DNASE1L3-deficient mice

Liangchen Lei, Jiaxiu Yu, Bo Zhang, Pengpeng Liu, Zhuo Meng, Youai Song, Jianwei Lan, Binjie Li, and Quanyan Liu

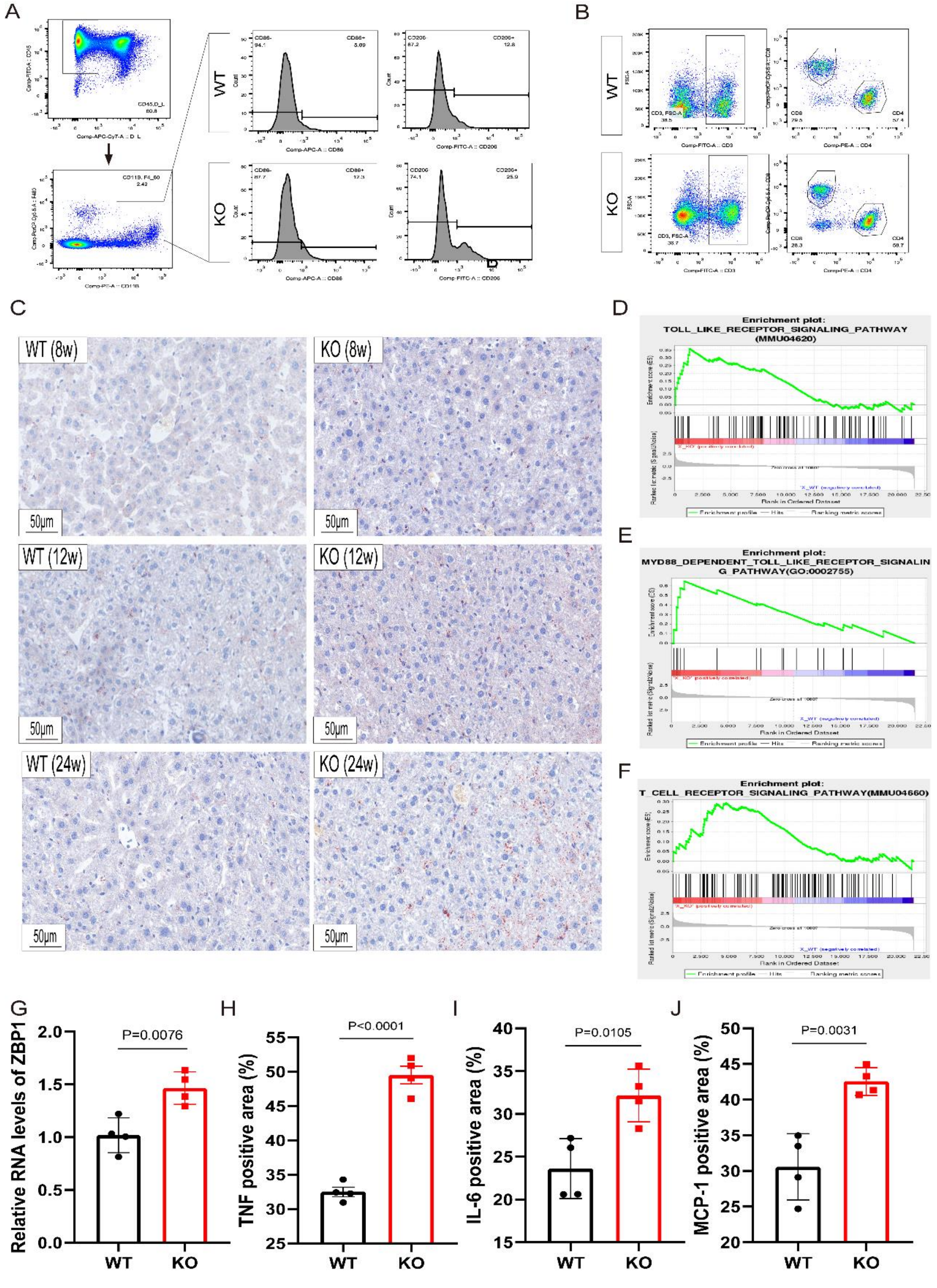

A
B
WT
KO
CD86-
CD86+
CD206-
CD206+
Comp-APC-A :: CD86
Comp-FITC-A :: CD206
Comp-FITC-A :: CD3
Comp-PE-A :: CD4
C
WT (8w)
KO (8w)
WT (12w)
KO (12w)
WT (24w)
KO (24w)
50μm
D
Enrichment plot:
TOLL_LIKE_RECEPTOR_SIGNALING_PATHWAY
(MMU04620)
E
Enrichment plot:
MYD88_DEPENDENT_TOLL_LIKE_RECEPTOR_SIGNALIN
G_PATHWAY(GO:0002755)
F
Enrichment plot:
T_CELL_RECEPTOR_SIGNALING_PATHWAY(MMU04660)
Rank in Ordered Dataset
Enrichment profile
Hits
Ranking metric scores
G
Relative RNA levels of ZBP1
P=0.0076
H
TNF positive area (%)
P<0.0001
I
IL-6 positive area (%)
P=0.0105
J
MCP-1 positive area (%)
P=0.0031
WT
KO

Supplementary Figure S1. DNASE1L3 deficiency skews macrophage polarization and activates inflammatory signaling pathways.

(A) Flow cytometric analysis of splenic macrophages. $CD45^+CD11b^+F4/80^+$ cells were gated, and polarization was assessed by CD86 (M1 marker) and CD206 (M2 marker) expression. KO mice showed an increased proportion of $CD86^+$ M1-like macrophages and a reduced proportion of $CD206^+$ M2-like macrophages compared with WT controls.

(B) Flow cytometric analysis of splenic T lymphocyte subsets. $CD3^+$ T cells were gated, and further subdivided into $CD4^+$ and $CD8^+$ populations. No significant differences were observed in the $CD4^+/CD8^+$ distribution between WT and KO groups.

(C) Representative Oil Red O staining of liver sections from WT and KO mice at 8, 12, and 24 weeks of age(scale bar: 50 μm).

(D–F) Gene Set Enrichment Analysis (GSEA) identified significant enrichment of inflammatory signaling pathways in KO livers, including (D) Toll-like receptor signaling (NES = 1.56, nominal P < 0.001, FDR q = 0.43), (E) MyD88-dependent Toll-like receptor signaling (NES = 1.63, nominal P = 0.004, FDR q = 0.30), and (F) T cell receptor signaling (NES = 1.32, nominal P < 0.001, FDR q = 0.49).

(G) Quantitative RT-PCR analysis showing elevated hepatic mRNA levels of Zbp1 in KO mice compared with WT controls (P = 0.0076; n = 4 per group).

(H–J) Quantification of IHC staining showing significantly higher hepatic expression of proinflammatory cytokines in KO mice, including (H) TNF (P < 0.0001), (I) IL-6 (P = 0.0105), and (J) MCP-1 (P = 0.0031) (scale bar, 50 μm; n = 4 per group).

Data are presented as mean ± SEM. Statistical comparisons were performed using unpaired two-tailed Student's t-test. Exact P values are indicated; P < 0.05 was considered statistically significant.

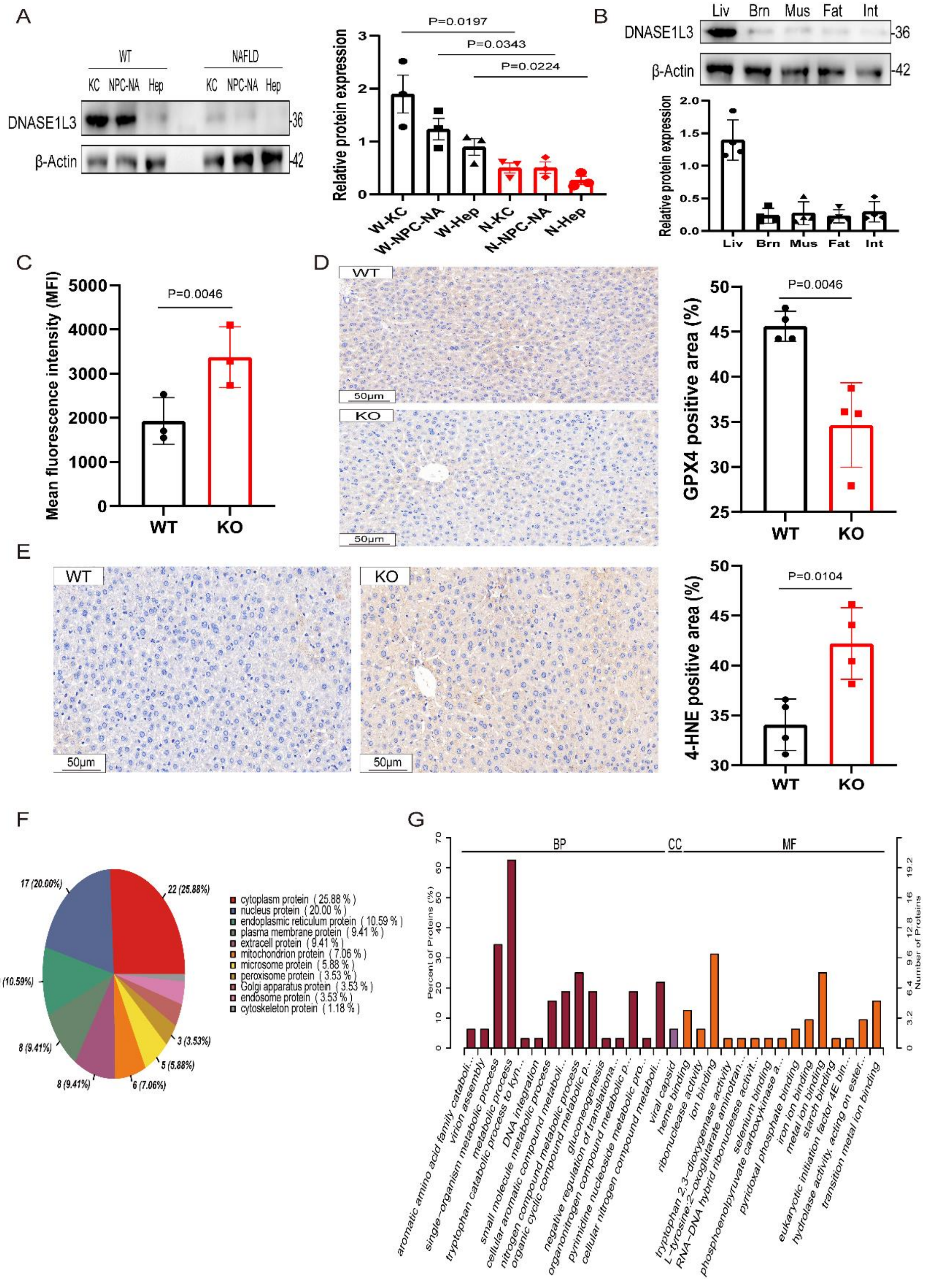
A
WT
NAFLD
KC NPC-NA Hep
KC NPC-NA Hep
DNASE1L3
β-Actin
36
42
P=0.0197
P=0.0343
P=0.0224
Relative protein expression
W-KC
W-NPC-NA
W-Hep
N-KC
N-NPC-NA
N-Hep
B
Liv Brn Mus Fat Int
DNASE1L3
β-Actin
36
42
Relative protein expression
C
P=0.0046
Mean fluorescence intensity (MFI)
WT
KO
D
WT
KO
50μm
P=0.0046
GPX4 positive area (%)
E
WT
KO
50μm
P=0.0104
4-HNE positive area (%)
F
22 (25.88%)
17 (20.00%)
9 (10.59%)
8 (9.41%)
8 (9.41%)
6 (7.06%)
5 (5.88%)
3 (3.53%)
cytoplasm protein (25.88 %)
nucleus protein (20.00 %)
endoplasmic reticulum protein (10.59 %)
plasma membrane protein (9.41 %)
extracell protein (9.41 %)
mitochondrion protein (7.06 %)
microsome protein (5.88 %)
peroxisome protein (3.53 %)
Golgi apparatus protein (3.53 %)
endosome protein (3.53 %)
cytoskeleton protein (1.18 %)
G
BP
CC
MF
Percent of Proteins (%)
Number of Proteins
aromatic amino acid family cataboli...
virion assembly
single-organism metabolic process
metabolic process
tryptophan catabolic process to kyn...
DNA integration
small molecule metabolic process
cellular aromatic compound metaboli...
nitrogen compound metabolic process
organic cyclic compound metabolic p...
gluconeogenesis
negative regulation of translationa...
organonitrogen compound metabolic pro...
pyrimidine nucleoside metabolic pro...
cellular nitrogen compound metaboli...
viral capsid
heme binding
ribonuclease activity
ion binding
tryptophan 2,3-dioxygenase activity
L-tyrosine:2-oxoglutarate aminotran...
RNA-DNA hybrid ribonuclease activit...
selenium binding
phosphoenolpyruvate carboxykinase a...
pyridoxal phosphate binding
iron ion binding
metal ion binding
starch binding
eukaryotic initiation factor 4E bin...
hydrolase activity, acting on ester...
transition metal ion binding

Supplementary Figure S2. DNASE1L3 deficiency induces hepatic oxidative stress, ferroptosis, and metabolic remodeling.

(A) Western blot analysis of DNASE1L3 protein expression in Kupffer cells (KC), non-parenchymal non-adherent cells (NPC-NA), and hepatocytes (Hep) isolated from WT and NAFLD mice. Quantification showed significantly reduced DNASE1L3 levels in NAFLD hepatic cell subsets (P = 0.0197 for WT-KC vs NAFLD-KC; P = 0.0343 for WT-NPC-NA vs NAFLD-NPC-NA; P = 0.0224 for WT-Hep vs NAFLD-Hep; n = 3).

(B) Western blot analysis of DNASE1L3 expression across major metabolic organs, including liver (Liv), brain (Brn), muscle (Mus), adipose tissue (Fat), and intestine (Int), showing the highest expression in the liver (n = 3).

(C) Flow cytometric quantification of hepatic reactive oxygen species (ROS) levels expressed as mean fluorescence intensity (MFI). KO mice exhibited significantly elevated ROS levels compared with WT controls (P = 0.0046; n = 3).

(D) Representative immunohistochemical (IHC) staining of GPX4 in liver sections from WT and KO mice (left). Quantification revealed reduced GPX4-positive area in KO livers compared with WT controls (P = 0.0046; scale bar, 50 μm; n = 5).

(E) Representative IHC staining of 4-hydroxynonenal (4-HNE), a marker of lipid peroxidation, showing increased staining intensity and positive area in KO livers compared with WT controls (P = 0.0104; scale bar, 50 μm; n = 5).

(F) Subcellular distribution of DEPs identified in liver tissues from WT and KO mice. A total of 85 DEPs were annotated based on UniProt and mainly localized to the cytoplasm (25.88%), nucleus (21.18%), ER (10.59%), plasma membrane (9.41%), as well as mitochondria, microsomes, and Golgi apparatus, suggesting widespread disturbance of organelle-specific homeostasis.

(G) GO enrichment analysis of upregulated DEPs across three categories: biological process (BP), cellular component (CC), and molecular function (MF). Enriched pathways included lipid metabolic processes, metal ion transmembrane transport, and redox-related functions, reflecting broad hepatic metabolic perturbation.

Data are presented as mean ± SEM. Statistical comparisons were performed using unpaired two-tailed Student's t-test. Exact P values are indicated; P < 0.05 was considered statistically significant.DEPs were defined by fold change > 1.5 and P <0.05. Subcellular localization was assigned using the UniProt Knowledgebase. GO enrichment was performed using the DAVID bioinformatics tool with a significance threshold of P <0.05.

Supplementary Table 1. Oligonucleotide sequences used in this study

**Name**	**Sequence**
Atf4	Forward: 5'- ATGGCCGGCTATGGATGAT -3'<br>Reverse: 5'- CGAAGTCAAACTCTTTCAGATCCATT -3'
Atf6	Forward: 5'- GACGAGGTGGTGTCAGAG -3'<br>Reverse: 5'- GACAGCTCTTCGCTTTGGAC -3'
Chop	Forward: 5'- CTGGAAGCCTGGTATGAGGAT -3'<br>Reverse: 5'- CAGGGTCAAGAGTAGTGAAGGT -3'
Xbp1 spliced	Forward: 5'- AAGAACACGCTTGGGAATGG -3'<br>Reverse: 5'- CTGCACCTGCTGCGGAC -3'
Tlr4	Forward: 5'- AGCTTCTCCAATTTTTCAGAACTTC -3'<br>Reverse: 5'- TGAGAGGTGGTGTAAGCCATGC-3'
Nlrp3	Forward: 5'- TCACAACTCGCCCAAGGAGGAA -3'<br>Reverse: 5'- AAGAGACCACGGCAGAAGCTAG-3'
Zbp1	Forward: 5'- GATCTACCACTCACGTCAGGAAG -3'<br>Reverse: 5'- GGCAATGGAGATGTGGCTGTTG-3'
Dnase1l3 gRNA	Forward strand of gene: GGTGTGAGCCCCTAGAGTTTGGG<br>Reverse strand of gene: TGGAGAAGGCTGTTACACTATGG
Genotyping primers for WT allele (800 bp)	Forward 5'- CAGTGAAGGAATAGGAGACT -3'<br>Reverse 5'- TGCTTAGAGTGCTCATCC -3'
Genotype Identification KO (318 bp)	Forward 5'- CAGGACTGGATAATGTACTTAG -3'<br>Reverse 5'- TGTGGAGAAGGCTGTTAC -3'

Supplementary Table 2. Functional classification of genes in chromosome 4 high-mutation region

Functional Category	Gene Count	Gene Symbol	Full Gene Name
Antioxidant Defense	3	Hmox1, Nqo1, Slc7a11	Heme oxygenase 1; NAD(P)H quinone dehydrogenase 1; Solute carrier family 7 member 11
Antioxidant/Ferroptosis	1	Gpx4	Glutathione peroxidase 4
Antiviral Response	2	Ifnb1, Irf7	Interferon beta 1; Interferon regulatory factor 7
Autophagy	1	Atg5	Autophagy related 5
DNA Sensing	2	Trex1, Zbp1	Three prime repair exonuclease 1; Z-DNA binding protein 1
Energy Metabolism	2	Ppargc1a, Slc2a1	Peroxisome proliferator-activated receptor gamma coactivator 1-alpha; Solute carrier family 2 member 1
ER Stress	2	Atf6, Chop	Activating transcription factor 6; DNA damage-inducible transcript 3 (CHOP)
Ferroptosis	1	Acsl4	Acyl-CoA synthetase long-chain family member 4
Inflammatory Response	6	Ccl2, Cxcl10, Il1b, Il6, Ptgs2, Tnf	C-C motif chemokine ligand 2; C-X-C motif chemokine ligand 10; Interleukin 1 beta; Interleukin 6; Prostaglandin-endoperoxide synthase 2 (COX-2); Tumor necrosis factor
Iron Homeostasis	4	Fth1, Ireb2, Slc25a37, Tfrc	Ferritin heavy chain 1; Iron responsive element binding protein 2; Solute carrier family 25 member 37 (Mitoferrin-1); Transferrin receptor
Lipid Metabolism	6	Acaca, Acot7, Etnk2, Etnk3, Fasn	Acetyl-CoA carboxylase alpha; Acyl-CoA thioesterase 7; Ethanolamine kinase 2; Ethanolamine kinase 3; Fatty acid synthase
Signal Transduction	2	Mavs, Stat1	Mitochondrial antiviral-signaling protein; Signal transducer and activator of transcription 1
Transcriptional Regulation	1	Usf1	Upstream transcription factor 1
Viral Sensing	1	Ddx58	DExD/H-box helicase 58
